# Case report and literature review of small bowel obstruction secondary to gastric band tubing

**DOI:** 10.1097/RC9.0000000000000409

**Published:** 2026-04-01

**Authors:** Konstantinos Mouskas, Rollin William Johnson, Vaughn Sherman, Suman Paritosh

**Affiliations:** Department of General Surgery, Wyckoff Heights Medical Center, Brooklyn, NY, USA

## Abstract

**Introduction and Importance:**

Laparoscopic adjustable gastric banding (LAGB) is a surgical intervention for treating morbid obesity. It is not without its complications and, very rarely, can cause small bowel obstruction (SBO) from the gastric banding tubing interacting with the small bowel.

**Case Presentation:**

This case report showcases a rare SBO in a 45-year-old woman who underwent LAGB 5 years prior. Radiological evaluation by CT scan showed dilated proximal bowel loops with air-fluid levels and an abrupt change in caliber in the mid-abdomen, concerning for small bowel obstruction. The patient then underwent a diagnostic laparoscopy, which revealed gastric band tubing looping around the common channel, causing a SBO. The gastric band connection tubing was severed to resolve the obstruction.

**Clinical Discussion:**

SBO secondary to LAGB is a very rare complication. Literature review yielded 17 previous cases with various outcomes. Ten required removal of the gastric band tubing, four had the hernia reduced without removal of the tubing or gastric band, and three required bowel resection. When a patient presents with an SBO in the setting of a LAGB, it is important to consider the tubing as a possible cause of an internal hernia.

**Conclusion:**

While internal herniations represent a more common complication of Roux-en-Y Gastric Bypass in literature, bariatric surgeons should also consider it after a LAGB procedure.

## Introduction

Obesity has approached an epidemic proportion in the United States, posing an increased burden on our health care system while also putting patients at risk of increased morbidity, mortality, and incommensurable economic costs. Morbid obesity affects 9.4% of people in the United States^[^1^]^ and approximately 0.5% of these people elect to undergo bariatric surgery as part of treatment^[^2^]^. Laparoscopic bariatric surgery has become the frontier of surgical interventions that target weight loss in patients with a body mass index (BMI) ≥ 40 kg/m^2^ or a patient that is ≥ 35 kg/m^2^ with accompanying comorbidity. Laparoscopic bariatric surgery offers durable results for sustained weight loss and improvement of obesity-associated comorbidity^[^3^]^.

Amongst laparoscopic options, currently the three most common procedures used for weight loss are Sleeve Gastrectomy (SG), Roux-en-Y Gastric Bypass (RYGB), and Biliopancreatic Diversion with Duodenal Switch (BPD-DS/SADI-S)^[^4^]^. During the mid-2000s, Adjustable Gastric Banding made 30%–40% of all bariatric surgeries, but due to high complication rates and suboptimal weight loss it is now reported as <1% used in surgical practices^[^4^]^.

HIGHLIGHTS

- Case report of gastric band tubing causing an small bowel obstruction and management
- Literature review of all previous similar case reports and discussion and table presentation

The AGB procedure consists of an inflatable silicone band that wraps around the upper stomach. A small stomach pouch is then created, which limits the amount of space available for food, leading to early satiety and subsequent weight loss. Patients have the ability to control the levels of band restriction by a subcutaneous port allowing for gradual regulation of satiety and lower perioperative morbidity^[^5^]^.

The Lap-Band may present with complications such as band slippage (gastric prolapse), pouch or esophageal dilation, device erosion, port-site issues, and variable weight loss outcomes^[^5^]^. In this case report, we present the complication of small bowel obstruction (SBO) secondary to gastric band connection tubing wrapping around the transition point of the ilium. This rare complication has only ever been described in case reports, with 17 cases being discovered on literature review. Review of these case reports showed many different possible presentations and results of this pathology, leading the authors to believe that comparisons and a summary of these presentations would be beneficial as well.

This case report has been reported in line with the scare checklist^[^6^]^.

## Case presentation

A 45-year-old woman with a history of Roux-En-Y in 2009 and a gastric band placement in 2019 presented to the emergency department complaining with abdominal pain, nausea, and vomiting after drinking beer the night before. She had not passed gas or stool for 24 hours. On exam the patient was tender to palpation throughout with mild distention. Her labs were notable for a lactic acidosis of 3.3 mmol/L and an ethanol level of 139 mg/dL.

**Figure 1.**
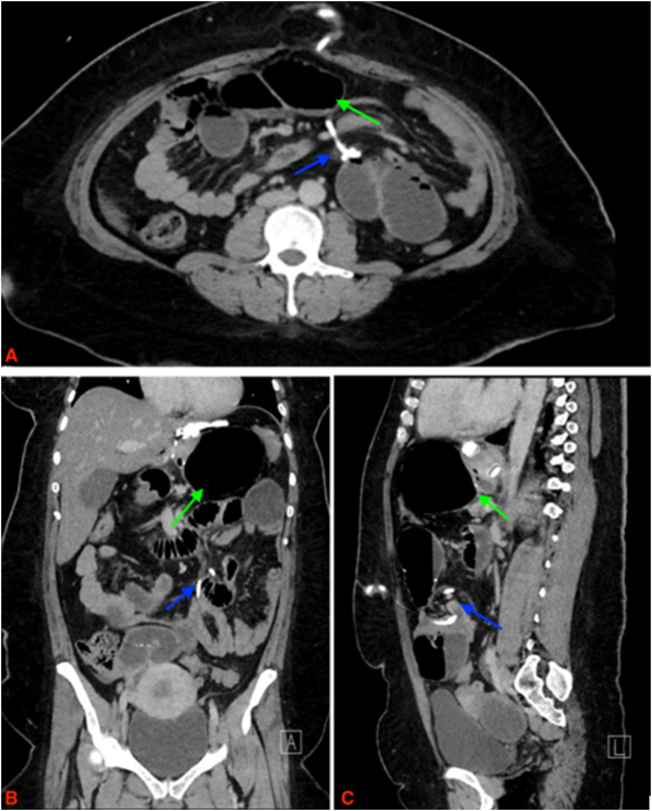
(A) Axial CT scan; (B) sagittal CT scan; (C) coronal CT scan. Imaging of the abdomen and pelvis on admission depicting the coiling of the gastric connecting tube around the transition point of the SBO (blue arrow) and the dilated small bowel segments (green arrow). No closed-loop internal hernia was identified on this scan, which was later seen intraoperatively.

**Figure 2.**
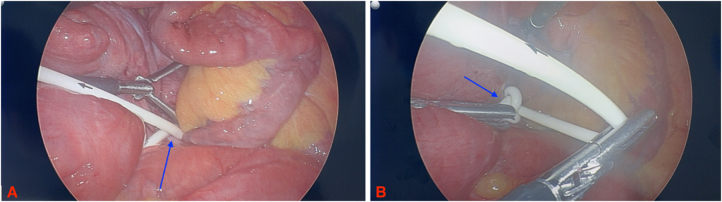
Connecting tube coiled tightly around the ileum causing a blind loop obstruction.

Due to concern for SBO, the patients received a CT scan of the abdomen and pelvis, which revealed a SBO and coiling of the gastric connecting tube around the transition point of the SBO (Fig. 1). The patient was immediately taken to the operating room (OR) for diagnostic laparoscopy. Intraoperatively, all anastomoses were seen to be intact and healthy. The connecting tube was traced from the gastric band and found to be coiled tightly around the ileum causing a closed loop obstruction (Fig. 2). Attempts at reducing the obstruction failed, ultimately requiring the connection tubing to be divided and extracted from the abdomen. The gastric band was allowed to remain. The abdomen was then desufflated, and all port sites were closed in a subcuticular fashion.

Overnight the lactic acidosis resolved to 0.8 mmol/dL, and the patient was aggressively hydrated. On postoperative day 2, the patient was having bowel function and tolerating a diet and was cleared for discharge. She later was seen in her 2-week follow-up and appeared to be healing well. Upper endoscopy in the outpatient setting revealed a gastric pouch and a healthy gastrojejunostomy anastomosis at 4 weeks postoperatively. The patient was then taken for laparoscopic gastric band removal and recovered without any complications 2 months after initial presentation.

## Discussion

The Adjustable Gastric Band procedure has been used as a minimally invasive surgical weight loss intervention since 1993 but progressively dropped in popularity due to increased postoperative complications^[^5^]^. There is a reported reoperation rate of 40%–60% at 10–15 years postoperatively mainly due to band slippage (5-20%), band erosion (1%–10%) and tubing related complications (tubing kink, disconnection)^[^7,8^]^. SBO is a rare (<0.5%)^[^9,10^]^, yet serious complication of LAGB that has been reported infrequently in case reports. Our case presents a unique patient who had received a Roux-en-Y Gastric Bypass, followed by an LAGB. RYGB-associated SBOs have been widely reported in literature via both case reports and systematic reviews, such as the ones of Murakami *et al* and Elms *et al*^[^11,12^]^.

A literature review was performed to identify previous case reports that described LAGB tubing causing SBO. This was done through searching on Pubmed for all case reports related to gastric banding which resulted in 336 articles. Two authors then opened and read each case report to determine if it met requirements to be listed within this manuscript. Both authors needed to agree in order for it to be included. This process yielded 17 case reports from the last 30 years.

When a patient with a previous history of bariatric surgery presents with signs of SBO, the concern is internal hernia and structural integrity of the anastomosis^[^13,14,15^]^. It is because of this that patients with RYGB will be taken directly to the OR when they present with SBO on CT scan. This allowed for early identification of the cause of the SBO via laparoscopy. We had theorized that patients that have LAGB-associated SBO without RYGB would likely to have worse outcomes due to the decreased urgency for operative treatment; however, it does appear that most cases of SBO caused by gastric band connection tubing are successfully managed without resection of small bowel. In our literature review, we only found three cases in which resection was needed^[^16,18^]^. Our review findings were composed of 17 LAGB-associated SBO cases. Our case was one of two identified with a concurrent Roux-en-Y gastric bypass. The most common surgical intervention for SBO secondary to gastric band tubing was band removal, which resulted in resolution of the obstruction (Table 1). There were four cases in which the gastric band and the tubing were allowed to remain after reduction of the hernia.

**Table 1:** Literature review of LAGB and SBO with clinical presentation. (NR: not reported).

Article	Years to event	Age/sex	BMI kg/m	Presentation	Treatment
Sharma K. *et al*^[^9^]^	4	46/F	39.06	Nausea, bilious vomiting, and abdominal pain, no bowel movements for 3 days	Exploratory laparoscopy into an exploratory laparotomy – tube disentanglement
Abeysekera A *et al*^[^19^]^	15	43/F	45	Acute abdominal pain leading to acute SBO with focal perforation	Laparotomy – band/tube removal
Suter KJ *et al*^[^10^]^	2	52/F	NR	Abdominal pain, vomiting, loose stools	Laparoscopy – division of adhesion, band/tube removal
Bassam A^[^20^]^	8	54/F	41.2	Abdominal pain, abdominal distention	Incisional drainage and access-port removal, 3 mo later: gastric band removal
Quarto G. *et al*^[^21^]^	2	47/F	NR	Nausea, vomiting, leukocytosis, distended abdomen, generalized tenderness	Band removal
Tan LB *et al*^[^22^]^	5	47/F	NR	Severe colicky pain, profuse vomiting, abdominal distention	Connecting tube removal
Nasser H. *et al*^[^23^]^	7	43/M	NR	Epigastric abdominal pain, vomiting, cholestatic labs	Band removal through enterotomy
Oppliger F. *et al*^[^24^]^	7	43/M	NR	Epigastric and periumbilical region pain, nausea	Laparoscopy – tube shortening
Shipkov CD *et al*^[^16^]^	9	42/F	43	Acute abdomen	Bowel resection, terminal ileostomy
Zappa MA *et al*^[^25^]^	3	61/F	40	Recurrent abdominal pain	Band freed from bowel and allowed to remain
Bueter M^[^26^]^	1.1	65/M	NR	Recurrent vomiting, epigastric pain	Band removal via jejunotomy
Sleiman A *et al*^[^8^]^	10	69/F	31.6	Colicky epigastric pain, abdominal distention, vomiting	Endoscopic removal of gastric band
Lemaire J *et al*^[^27^]^	10	50/F	55	Acute abdominal pain located in the left flank, nausea, vomiting and ileus	Exploratory laparotomy – band/tube removal
Salar O *et al*^[^28^]^	5	46/F	140 kg	Nausea, colicky upper abdominal pain, fever, and bilious vomiting	Enterotomy – band/tube Removal
Agahi A *et al*^[^17^]^	2	56/F	NR	Intermittent nausea and abdominal pain	Cecal volvulus segment resected with band removal
Ng M *et al*^[^18^]^	2	67/F	NR	Abdominal pain and distension	Right hemicolectomy and gastric tubing sutured to the peritoneum
Strobos E *et al*^[^29^]^	1	44/M	NR	Abdominal pain and distension	Tubing freed from bowel and sutured to peritoneum

In our case of SBO secondary to a LAGB, we were able to swiftly surgically manage due to the fear of internal herniation. For patients without RYGB, it would be easy to initially manage these patients conservatively as LAGB connection tubing is such a rare lead point for SBO. The delay in surgical care could theoretically lead to worse surgical outcome and need for resection of bowel. We believe this is not reflected in the literature because a patient who has received a LAGB would already be under the care of a surgeon who placed the medical device. Patients who present with SBO after any bariatric surgery should be treated immediately with bowel rest and nasogastric tube (NGT) decompression. With LAGB, patients should have the gastric band desufflated in conjunction with NGT decompression^[^30^]^.

Two methodologies of preventing this pathology have been described. The first is to prevent a large length of redundant tubing in the abdomen on application. In the case reports listed below, many authors came to the conclusion that the increased length allowed for the twisting of the bowel around the tubing^[^16–18,25^]^. The treatment after reducing the SBO has been to tack the redundant band to the peritoneum. The second is to make sure there is adequate coverage of the bowel with the omentum on LAGB application^[^29^]^. The omentum will act as a barrier that will prevent the gastric band tubing from interacting with the bowel.

While LAGB has fallen out of favor in the current literature, there is still a prevalent patient population with these devices in place. We have found that a SBO secondary to gastric band tubing can present as far out as a decade from initial surgery with the longest duration being 15 years. It is important to keep in mind that an SBO in the presence of LAGB may be more insidious in nature as the presentation may not always be obvious^[^17,25^]^. Increased awareness of these rare complications is important when treating a patient that has SBO in the setting of LAGB.

## Conclusion

Long-term weight control is essential to the management of morbid obesity. Out of all the modern laparoscopic interventions, Adjusted Gastric Banding has lost its prevalence as the frontier of minimally invasive approaches due to the numerous complications that patients experience postoperatively. In this case report, we presented the case of an internal herniation that led to an obstructed small bowel 6 years after the placement of LAGB. This was a unique presentation due to previous RYGB. A literature review compared previous presentations, treatments, and outcomes of previous case reports. These authors would recommend early aggressive diagnostic testing, including diagnostic laparoscopy, on all patients with SBO and previous bariatric surgery, including gastric banding.

Generative AI was not used in the writing process of this manuscript.

## Data Availability

All available data can be made available to other researchers pending approval of a research proposal by the Wyckoff Heights study team. Researchers can contact the corresponding author to make such a request.
